# Use of extensively hydrolysed formula for refeeding neonates postnecrotising enterocolitis: a nationwide survey-based, cross-sectional study

**DOI:** 10.1136/bmjopen-2015-008613

**Published:** 2016-07-07

**Authors:** Alexandre Lapillonne, Maroun Matar, Ariane Adleff, Marwa Chbihi, Elsa Kermorvant-Duchemin, Florence Campeotto

**Affiliations:** 1 Neonatal Department, APHP Necker-Enfants Malades Hospital, Paris, France; 2 Paris Descartes University, Paris, France; 3 Neonatology Division, University Medical Center of Rizk Hospital, Rizk, Lebanon; 4 Private Practice, Pertuis, France; 5 Pediatric Gastroenterology Department, APHP Necker-Enfants Malades Hospital, Paris, France

**Keywords:** NEONATOLOGY, hydrolyzed formula, low birth weight infants, necrotizing enterocolitis, NUTRITION & DIETETICS

## Abstract

**Objective:**

To evaluate the prevalence of and reasons for using extensively hydrolysed formulas (EHFs) of cow's milk proteins in the French neonatal units as well as the modality of their prescription for refeeding infants recovering from necrotising enterocolitis (NEC).

**Methods:**

A multicentre nationwide cross-sectional study using a questionnaire to address the prevalence of use and the reasons for prescribing EHF in hospitalised neonates and to examine the protocols and the actual reasons for their use for refeeding infants in recovery from NEC. The questionnaire was sent to only 1 senior neonatologist in each neonatal unit included in the study.

**Results:**

More than half of the French neonatal units participated in the survey. 91% of the surveyed units used EHF. Of 1969 infants hospitalised on the day the survey was run, 12% were fed on an EHF. 11% of the EHF prescriptions were due to previous NEC. The main reasons for using an EHF to feed infants post-NEC were the absence of human milk (75%) and surgical management of NEC (17%). When given, EHF was mainly prescribed for a period varying between 15 days and 3 months. None of the involved units continued using the EHF after 6 months of age. More than half of the surveyed units acknowledged hospitalising infants for the initiation of weaning EHF but only 21% of them tested these infants for cow's milk allergy.

**Conclusions:**

The prevalence of EHF use in the French neonatal units is high. Refeeding infants post-NEC is one of the main reasons for such a high prevalence. The main incentive for using an EHF is the absence of human breast milk, either maternal or donor.

**Strengths and limitations of this study**

- This nationwide survey shows for the first time that the prevalence of use of extensively hydrolysed formula (EHF) of cow's milk proteins is high in the French neonatal units.
- Refeeding infants after necrotising enterocolitis is one of the main reasons for prescribing EHF for preterm infants, especially when maternal or donor breast milk is not available.
- The weaning modalities of EHF varied between the units surveyed, signifying a considerable lack of consensus.
- This is a questionnaire-based clinical practice survey.
- The benefit–risk ratio of the EHF use, as well as the modality for their weaning, needs to be evaluated by more studies.

## Introduction

Necrotising enterocolitis (NEC) is a major concern in preterm, especially extremely preterm (<28 weeks' gestation), neonates worldwide. Its mean prevalence among very preterm infants is about 7% with a reported mortality rate of 20–30%.^1^ Many clinical trials have evaluated the safety and benefits of preventive strategies, while others have attempted to determine the best possible medical or surgical management.^2^ ^3^

In contrast, there is a perceived lack of consensus on when and how enteral feeding should be reintroduced, and advanced till achieving the target volumes.^4^ The choice of post-NEC feeding remains controversial. In most instances, when available, maternal breast milk is considered the optimal feeding.^5^ In case of non-availability, some neonatologists use preterm milk formula, provided that the gastrointestinal injury is limited. Some others would use either donor breast milk or hydrolysed formulas. When the gastrointestinal injury is substantial, elemental or lactose-free hydrolysed formulas with variable content of medium-chain triglycerides are used to get over the problem of malabsorption.^5^

The purpose of this study was to evaluate the prevalence and indications of use of extensively hydrolysed formula (s) (EHF) of cow’s milk proteins in the French neonatal units and to examine the protocols guiding their use for refeeding infants post-NEC.

## Materials and methods

To conduct this study on a nationwide level, we ran a survey using a questionnaire especially designed to investigate routine feeding practices in the involved neonatal units. The survey used the technique of a closed-answer questionnaire to limit the variability of answers and decrease the number of incomplete answers, focusing on enteral feeding practices postmedical or surgical NEC (questionnaire available on demand).

The first series of questions aimed at determining the prevalence and indications of EHF use in neonates. To achieve this, we ran a multicentre nationwide cross-sectional study, and requested the neonatologists responding to the questionnaire to report the total number of infants actually hospitalised in their units on the day the questionnaire was filled in, together with the main reported indications of EHF. Only infants who had NEC of grade II or III were considered for the study, as grade I NEC can be confused with other causes of feeding intolerance.

The second series of questions focused on the feeding protocols and reasons for EHF use when prescribed for refeeding infants recovering from NEC of stage II or III. More specifically, questions targeted the duration of use, and the protocol used for weaning infants of EHF to regular cow's milk formula or human milk. It should be noted that the medical decision to use an EHF in France, particularly during hospitalisation, was not made under any financial pressure, conflict of interest or mitigation as every legal resident of France, including preterm infants, has, by law, a full universal coverage of healthcare.

The exhaustive list of neonatal units of metropolitan France and overseas territories was established by combining the lists of the national scientific societies involved in neonatal care and those of all the regional healthcare services. Neonatal units having a high acuity or intensive care beds were selected for the study. Only one questionnaire per unit, accompanied by a cover letter and a reply envelope, was posted by mail to the head of the unit. He was asked to complete the survey questionnaire or to delegate the task to a colleague with more than 3 years of clinical experience in neonatal medicine, and more than 20% of time devoted to direct patient care.

In order to reduce the risk of selection bias, we aimed at surveying at least half of the nationwide neonatal units to have a picture of the nutritional care of the preterm infants post-NEC as clearly and as accurately as possible. A reminder letter was subsequently posted 1 month later to the non-responders to achieve our goal. The identity of the neonatologists contacted and requested to complete the survey remained blinded for the analysis.

### Statistical analysis

Statistical analyses were restricted to completed questionnaires with evaluable results. Data were analysed using Minitab 13.3 software (Minitab Inc, State College, Pennsylvania, USA). General frequency responses to all survey items were determined and then used to test for associations among the categorical variables. When needed, data were split to cross tabs with respect to various grouping variables. Comparisons were made by Pearson χ^2^ test of independence. A p value ≤0.05 was considered statistically significant.

## Results

### Characteristics of the units

The goal of surveying at least half of the French neonatal units was reached since we received 174 responses from the 296 units contacted (58.8%). The characteristics of the responding units are reported in table 1. Of the 174 units surveyed, 158 (91%) routinely used EHF (table 1).

**Table 1:** Characteristics of the responding neonatal units

	Total
Units surveyed	
Number of units (n)	174
Number of units using EHF routinely (n)	158
Number of units routinely caring for infants post-NEC (n)	93
Number of admissions	
Number of admissions per year (n)*	61 578
GA<37 weeks (n)*	28 029
GA<28 weeks (n)*	2394

*Based on the responses of 150 units.

EHF, extensively hydrolysed formula; GA, gestational age; NEC, necrotising enterocolitis.

### Prevalence of use and reasons for feeding hospitalised neonates with EHF

Of the 1969 infants hospitalised on the day of the filling of the questionnaire, 238 (12.1%) received an EHF. The reasons for feeding hospitalised neonates with EHF were indicated in all cases as reported in table 2. Shortage of human milk is overall the main reason for prescribing EHF either for the initiation of feeding in preterm infants or for complementary feeding of breastfed infants. Among all the infants receiving EHF, 10.5% of the prescriptions were made because of a previous NEC.

**Table 2:** Prevalence of use and reasons for feeding hospitalised neonates with EHF

	n (%)
Infants hospitalised on the day of the questionnaire filling (n)	1969
Infants receiving an EHF, n (% of hospitalised infants)	238 (12.1%)
Reasons for feeding neonates with EHF	
Initiation of feeds	
After NEC stage II or III	25 (10.5%)
After perinatal asphyxia	8 (3.3%)
After any kind of surgery	4 (1.7%)
Shortage of human milk	
Feeding initiation of preterm infants in the absence of human milk	63 (26.5%)
Complementary feeding of breastfed neonates	84 (35.3%)
Allergy prevention in high-risk neonates	2 (0.8%)
Gastrointestinal symptoms (compatible or not with cow's milk protein allergy)	31 (13.0%)
Others (research protocol, hypoglycaemia, cholestasis, metabolic disease, etc, no reasons indicated)	21 (8.8%)

EHF, extensively hydrolysed formula of cow's milk proteins; NEC, necrotising enterocolitis.

### Nutritional protocols when using EHF for feeding infants recovering from NEC

Of the 174 units surveyed, 93 (53.4%) routinely took care of infants post-NEC. EHFs were routinely used in 88 (95%) of them (table 1).

The main reasons for using EHF as the preferred milk for feeding infants post-NEC were the absence of human breast milk (n=65/93, 75%) and when surgical management of NEC was required (n=15/93, 17%). The other reasons cited were NEC in term babies for whom EHF is nutritionally adapted, other associated digestive problems, NEC severity at onset and shortage of donor breast milk.

EHF, when given, was mainly prescribed for a period which varies from 15 days to 3 months. None of the units continued giving EHF after 6 months of age (table 3). The mode of weaning from EHF to regular cow's milk formula or donor breast milk is described in table 3. In the absence of breast milk, 83% of the units switched to a cow's milk formula, while others shifted to using either donor breast milk or a partially hydrolysed formula. Most of the surveyed units progressively weaned the EHF over a mean (±SD) period of 6.9 (±3.1) days. More than half of the units reported having the infants hospitalised for initiating the weaning process. However, only 21% of these units tested the infants for cow's milk allergy.

**Table 3:** Nutritional protocols of neonatal units using extensively hydrolysed formula (EHF) in preterm infants postnecrotising enterocolitis

Duration of EHF use	Per cent of units
<15 days	8
15–30 days	30
1–3 months	50
4–6 months	12
≥7 months	0
Weaning EHF in hospital	
Yes	52
No	48
Weaning EHF progressively over several days	
Yes	96
No	4
Weaning EHF after testing for cow's milk allergy	
Yes	21
No	79
Type of milk used for weaning EHF in the absence of mother's breast milk	
Donor breast milk	13
Regular cow's milk formula	83
Partially hydrolysed formula or other	7

## Discussion

To the best of our knowledge, this is the first study which attempts to determine the frequency of use of EHF in the neonatal units. We found that the prevalence of EHF use is high, approaching 12.1% of the enteral feeding prescriptions. The indications of EHF in neonates reported in the literature include absence of human milk, poor feeding tolerance,^6^ severe gastro-oesophageal reflux,^7^ ^8^ family history or clinical signs of cow's milk allergy,^9^ or history of gastrointestinal surgery or intestinal resection.^10^ Our study clearly shows that refeeding infants recovering from NEC is a frequent indication of EHF use in hospitalised neonates.

Most of the available literature focuses on the nutritional prevention, not the treatment of NEC. Furthermore, it is extremely vague regarding the timing of refeeding and the type of milk to be used after initial management or postoperatively for infants who have had NEC. Although it is well established that feeding should be suspended for a period of time that depends on the disease severity, there are no clear recommendations on when to restart feeding after the subsidence of the acute-stage NEC.^5^ ^11^ The choice of formula milk for refeeding infants post-NEC depends on many factors such as gestational age, the availability of human breast milk, risk of short gut syndrome and/or malabsorption, as well as risk of cow's milk allergy. Our study clearly shows that the main drive for choosing an EHF for refeeding NEC infants is the absence of human breast milk, either maternal or donor.

EHF is not the feeding formula usually cited in the few textbooks defining the feeding choices post-NEC.^5^ ^11^ There are, however, several putative reasons for choosing an EHF.

First, there is a debate on the direct contributory role of cow’s milk protein sensitisation in the pathogenesis of NEC.^12^ Cow's milk allergy is well recognised as a significant cause of morbidity in formula-fed term and recently in preterm infants.^13^ Several case reports have shown that cow’s milk protein allergy may be closely related to NEC,^14^ ^15^ and there is also evidence of in vitro sensitisation to cow's milk protein in peripheral blood mononuclear cells of preterm infants with NEC.^16^ ^17^ This suggests that cow’s milk intolerance should be evaluated when NEC occurs in case of absence of classical risk factors.^18^

Second, premature infants recovering from mucosal inflammation and prolonged periods of bowel rest are potentially at increased risk of antigenic response to intact proteins.^11^ It has been shown that allergy to cow's milk proteins in newborns who underwent gastrointestinal surgery is higher than expected in the absence of a family history of allergy.^19^ In this context, EHF may be useful for refeeding infants post-NEC. Nevertheless, no study until now has assessed the usefulness of such a strategy for possibly preventing cow's milk protein sensitisation.

Finally, the use of EHF may also be considered for their nutritional value. Indeed, they do not usually contain lactose and some of them do contain a significant amount of medium-chain triglycerides. These characteristics may improve absorption during refeeding, especially in surgical patients. Lactose is poorly tolerated in neonates with small bowel disease or resection because of the decrease in available mucosal lactase. Medium-chain triglycerides also improve fat absorption, known to be also reduced in cases of loss of absorptive area, rapid transit, bile acid depletion and/or bacterial overgrowth.^10^ In contrast, the theoretical advantage of hydrolysed over whole protein formulas of better absorption in case of a reduced absorptive area and decreased pancreatic enzyme output remains uncertain since it has been shown that dietary protein absorption capacity of the small intestine is normal for most neonates after intestinal surgery.^20^

If there are possible nutritional benefits for using EHF for feeding infants after NEC, they should be weighed against possible disadvantages.^21^ Indeed, these formulas have an energy density close to that of term formulas, in addition to usually low mineral and polyunsaturated fatty acid contents as compared with preterm formulas. Urinary nitrogen excretion is higher,^22^ and calcium and phosphorus absorption and nitrogen retention are lower in preterm infants fed with hydrolysed formula compared with those fed with whole protein formula.^23^ ^24^ These drawbacks may alter the quality of growth or decrease the lean body mass accretion in preterm infants receiving hydrolysed formula when compared with those receiving non-hydrolysed formula whether the growth rate was similar or not.^25^ ^26^

In the absence of specific recommendations or studies guiding or helping to assess the risk of food allergy in infants post-NEC, it is not surprising that our study shows a great heterogeneity in the weaning protocols of EHF. Interestingly, our study shows that the duration of use of EHF post-NEC is less than the 4–6 month period recommended by the guidelines for food allergy prevention.^27^ In addition to that, cow's milk proteins are frequently introduced without performing any appropriate diagnostic workup.

It should be recognised that our study has several limitations. It was performed in one country only and results may not be valid for other countries. It may be argued that this is a cross-sectional study that was performed at a single point of time. However, it is well known that such a study design is particularly suitable for assessing the prevalence of a disease or a treatment in a specific population.^28^ Although we aimed at and succeeded in assessing more than half of the French units, such a study design is prone to selection bias.^29^ We cannot therefore state that the non-responding units were those which used EHF less or more than the responding ones. Finally, this survey did not allow us to assess any longitudinal follow-up or incidence data of NEC.^28^ However, we were able to assess the intent-to-treat modalities of feeding infants post-NEC.

In conclusion, this study shows that the use of EHF in the neonatal units is frequent. Refeeding infants post-NEC is one of the reasons for such high prevalence. The main drive for using EHF is the absence of human breast milk, either maternal or donor. Patients with NEC represent a group of infants who may benefit from these EHF. However, the benefit/risk ratio of their use, as well as the modality of their weaning, needs to be further evaluated by more studies.
